# The Inositol Phosphate System—A Coordinator of Metabolic Adaptability

**DOI:** 10.3390/ijms23126747

**Published:** 2022-06-16

**Authors:** Becky Tu-Sekine, Sangwon F. Kim

**Affiliations:** 1 Department of Medicine, Division of Endocrinology, Diabetes and Metabolism, Johns Hopkins University, Baltimore, MD 21224, USA; btusekine@jhmi.edu; 2 Department of Medicine and Neuroscience, Division of Endocrinology, Diabetes and Metabolism, Johns Hopkins University School of Medicine, Baltimore, MD 21224, USA

**Keywords:** inositol polyphosphate, inositol pyrophosphate, IPMK, IP6K, AKT, PI3K, insulin, mitochondria, OXPHOS, glycolysis

## Abstract

All cells rely on nutrients to supply energy and carbon building blocks to support cellular processes. Over time, eukaryotes have developed increasingly complex systems to integrate information about available nutrients with the internal state of energy stores to activate the necessary processes to meet the immediate and ongoing needs of the cell. One such system is the network of soluble and membrane-associated inositol phosphates that coordinate the cellular responses to nutrient uptake and utilization from growth factor signaling to energy homeostasis. In this review, we discuss the coordinated interactions of the inositol polyphosphates, inositol pyrophosphates, and phosphoinositides in major metabolic signaling pathways to illustrate the central importance of the inositol phosphate signaling network in nutrient responses.

## 1. Introduction

All organisms depend on nutrients to generate the energy that sustains life, and the cellular programs required to adapt to nutrient availability and maintain intracellular energy are diverse and context dependent. This demands a coordinated series of sensors and effectors to monitor energy status and to adapt to nutrient availability. Such a signaling network must not only be complex and dynamic, but also tightly integrated to successfully prioritize and synergize multiple inputs to respond to rapid changes in the energy demand experienced by muscle and cardiac tissues under high workloads, to the rapid firing of neurons needed to create memories, or to the activation of immune cells to defend against viral infections. These and other events require carefully controlled changes in the use of glucose, fatty acids, and other nutrients to balance the need for rapid energy and biosynthetic precursors produced through glycolysis with the more efficient energy production achieved through oxidative phosphorylation. This integration results in the modulation of anabolic and catabolic pathways to ensure a balance between energy production and energy utilization. When key nutrient sensing pathways are disrupted, these signals become distorted and disease may ensue. Importantly, and perhaps surprisingly, most nutrient signaling converges on two primary metabolic nodes in mammalian cells: AMPK (AMP-activated protein kinase), which governs stress responses and the fasting state, and mTOR (mammalian target of rapamycin), which governs biosynthesis and the fed state. As may be predicted, the complete loss of either of these proteins results in embryonic lethality [1,2]. However, this deceptively simple platform is coordinated by the inositol polyphosphate network of enzymes, which integrates metabolic sensing from receptor activation to gene expression.

The inositol phosphate signaling network includes the celebrated phosphatidylinositol(4,5)bisphosphate and phosphatidylinositol(3,4,5)trisphosphate (hereafter referred to as PtdIns(4,5)P2 and PtdIns(3,4,5)P3) pathways, as well as the relative newcomers to metabolic control—the inositol polyphosphates (InsPs) and inositol pyrophosphates (PP-InsPs). The interconnected nature of the inositol phosphate biosynthetic pathways means that disruptions at almost any point can potentially create mass changes in both upstream and downstream pathway constituents, making it difficult to assign any observed phenotype with a single inositol phosphate component. This interconnectedness is dramatically illustrated under conditions of total inositol depletion, which results in the “inositol-less” death of cultured cells within hours [3]. Importantly, many key components and metabolic functions of the inositol system are conserved across eukaryotes, indicating that these molecules influence metabolism and energy homeostasis across the evolutionary timeline [4,5,6]. In this review, we examine the influence of the inositol phosphate system on the coordination of nutrient sensing and bioenergetic pathways to highlight its many roles in metabolic function and argue that this system should be viewed as a single network with respect to nutrient metabolism and bioenergetic homeostasis.

## 2. The Inositol Phosphate System

Appreciation of the inositol signaling system requires some familiarity with the basic components. This can be a bit of a hurdle as the compendium of inositol polyphosphates can appear complex and rather disorienting when viewed in its entirety. However, the initial effort pays dividends as this familiarity greatly increases one’s ability to grasp the potential impacts to signaling and bioenergetics that result from changes within this system. Detailed information on the nomenclature, stereochemistry, and chemical properties are readily available [7,8,9,10] and are only briefly covered here. The biological stability of D-myo-inositol (referred to as inositol hereafter) and the presence of six hydroxyls that can be rapidly phosphorylated in response to environmental cues and the needs of the cell make inositol ideally suited to act as a dynamic signal, producing what has been aptly described as an inositol polyphosphate code (“IP code”) [11]. The inositol polyphosphate pools and their modifying enzymes must therefore be connected to allow the rapid interconversion of one polyphosphate to another. How exactly these networks are connected in cells remains an area of many questions with few answers.

### 2.1. PtdInsPs, Ips, and PP-InsPs—Similar, But Not the Same

#### 2.1.1. PtdInsPs—The Phosphatidylinositol Lipids

**Figure 1:**
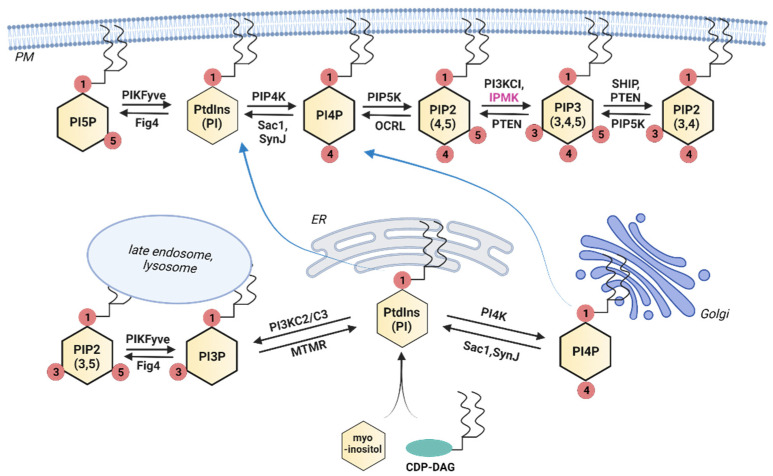
Biosynthesis of PtdInsPs in mammalian cells. The primary locations of the inositol lipids are shown along with the most common interconversions and converting enzymes. Blue arrows indicate the direction of lipid transport from one membrane compartment to another; black arrows indicate forward and reverse reactions. Note that PtdIns, the precursor to the PtdInsPs, is formed from myo-inositol and CDP-DAG at the ER (see text). ER: endoplasmic reticulum; CDP-DAG: cytidine diphosphate diacylglycerol; PIKFYVE: phosphoinositide kinase, FYVE-type zinc finger containing; FIG4: FIG4 phosphoinositide 5-phosphatase; PIP4K: phosphatidylinositol-5-phosphate 4-kinase; SAC1: SAC1 Like phosphatidylinositide phosphatase; SynJ: synaptojanin 1; PIP5K: phosphatidylinositol-4-phosphate 5-kinase; OCRL: OCRL inositol polyphosphate-5-phosphatase; PI3KC1: class I phosphatidylinositol 3-kinase; PTEN: phosphatase and tensin homolog; SHIP: SH2 domain-containing inositol 5′-phosphatase 1; PI3KC2/C3: class 2/class 3 phosphoinositide 3-kinase; MTMR: myotubularin-related protein/phosphatidylinositol-3-phosphatase. Created with BioRender.com, accessed on 27 May 2022.

Inositol phosphates can be separated into two general categories—those that are lipid-associated and those that are soluble. In mammals, the lipid-based group consists of seven members, each containing a phosphorylated inositol headgroup attached to (usually) a diacylglycerol (DAG) at the sn-1 position of the glycerol backbone. These are named according to the arrangement of the phosphate groups on the inositol ring. For example, PtdIns(4,5)P2 (aka PIP2) refers to the phosphatidylinositol lipid phosphorylated at the 4 and 5 positions of the inositol ring. We refer to these in general as phosphoinositides or PtdInsP. Due to their highly hydrophobic nature, these lipids are typically associated with various subcellular membranes, but they are also found in non-membranous nuclear subdomains [12,13]. The precursor lipid—PtdIns—is synthesized in the endoplasmic reticulum by the condensation of myo-inositol and cytidine diphosphate diacylglycerol (CDP-DAG) and is transported throughout the cell, where it acts as a structural membrane lipid as well as a PtdInsP precursor [14,15]. The inositol used in this reaction is often imported into the cell or recycled from PtdIns(4,5)P2, although it can also be synthesized from glucose [5,16,17]. A series of kinases and phosphatases transform PtdIns into PtdInsPs, as shown in Figure 1. PtdIns4P is primarily synthesized at the Golgi apparatus, both for use there and for transport to the plasma membrane for production of PtdIns(4,5)P2, and only a small percentage of PtdIns4P appears to be synthesized directly at the plasma membrane [18]. In contrast, PtdIns(4,5)P2 and PtdIns(3,4,5)P3 are primarily synthesized at the plasma membrane and are detected at low levels in other membranes throughout the cell.

#### 2.1.2. InsPs and PP-InsPs—The Inositol Polyphosphates and Inositol Pyrophosphates

**Figure 2:**
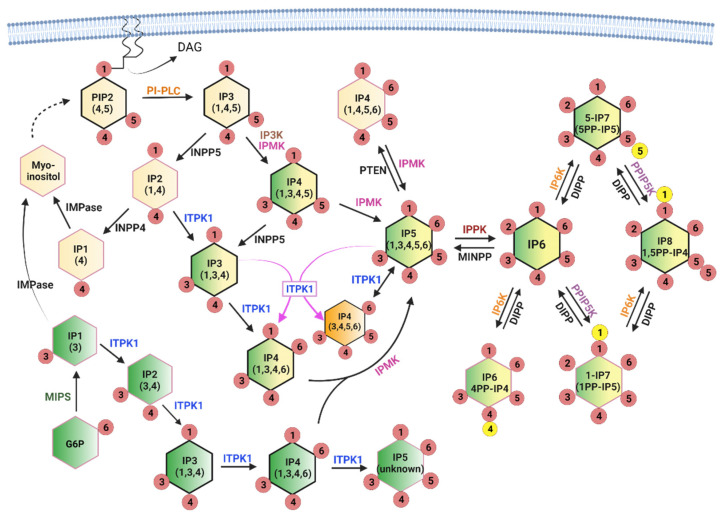
Biosynthesis of InsPs and PP-InsPs in mammalian cells. Lipid-derived precursors originate from PtdIns(4,5)P2 and are shown in yellow; glucose-derived precursors originate from G6P and are shown in green. Stereoisomers that can originate from both sources are shown as dual colors. InsP4(3,4,5,6) is highlighted in orange due to its unique function as an ion channel regulator (see text). Bold black outlines highlight the dominant synthetic pathways in humans. Forward and reverse reactions are indicated with black arrows; only the reverse reactions proposed to commonly occur are indicated. Magenta arrows indicate ITPK1 phosphotransferase reaction. The dashed arrow from myo-inositol to PIP2 refers to a multistep reaction. Kinases are color-coded to aid in visualization due to substrate promiscuity. Phosphatases are shown in black. PI-PLC: phosphatidylinositol specific phospholipase C; IP3K: inositol-trisphosphate 3-kinase; IPMK: inositol polyphosphate multikinase; ITPK1: inositol-tetrakisphosphate 1-kinase; IPPK: inositol-pentakisphosphate 2-kinase; IP6K: inositol hexakisphosphate kinase; PPIP5K: diphosphoinositol pentakisphosphate kinase; MIPS: myo-inositol 1-phosphate synthase; IMPase: inositol monophosphatase; INPP4/INPP5: inositol polyphosphate-4-phosphatase/inositol polyphosphate-5-phosphatase. Created with BioRender.com, accessed on 27 May 2022.

The soluble inositol phosphates contain only the inositol ring phosphorylated with one or more phosphates in various combinations. While this group has many more potential members than the lipid-based group, only a small subgroup is well-studied and known to be important for metabolic signaling. Inositol phosphates with single phosphates at any combination of positions on the inositol ring are often referred to as inositol polyphosphates (InsPs), while those with two phosphates at the same ring position (diphosphosphate) are referred to as inositol pyrophosphates (PP-InsPs). The PP-InsPs have been found to be particularly important in metabolic and bioenergetic processes [19,20]. The InsP/PP-InsPs are formed through two converging pathways, as shown in Figure 2. The first pathway is referred to as lipid-dependent and is initiated by the receptor-stimulated hydrolysis of PtdIns(4,5)P2, which releases Ins(1,4,5)P3. Ins(1,4,5)P3 is then dephosphorylated back to inositol for recycling into PtdIns or converted to higher IPs through a series of kinase reactions, with the outcome dependent on the competing enzyme activities that control degradation and synthesis [21,22].

The second metabolic pathway begins with the isomerization of glucose-6-phosphate (G6P) to inositol-3-phosphate (Ins3P) by myo-inositol 1-phosphate synthase (MIPS) [23]. As with Ins(1,4,5)P3, Ins3P can be dephosphorylated to inositol for other uses (including PtdIns synthesis) or converted to Ins(1,3,4)P3 for conversion to higher IPs through the Ins(1,4,5)P3 pathway [5]. There appears to be a preferred pathway for InsP synthesis in mammals defined, in part, by enzyme activities and substrate preferences, and these are indicated with black outlines in Figure 2. In addition, the contributing sources are color-coded to better illustrate the precursor identity. The stereoisomers that originate from glucose are represented in green, those from PtdIns(4,5)P2 are represented in yellow, and pools of stereoisomers that can originate from either branch are represented as dual colors (yellow and green). This makes it easy to appreciate that convergence of the two branches occurs at the level of Ins(1,3,4)P3, and that the glucose-dependent branch is capable of providing the same IPs as the lipid-dependent branch. The current consensus is that the lipid-dependent branch is the primary source of InsP metabolism under most conditions, although some metabolic crises (such as phosphate starvation) appear to stimulate the glucose-dependent branch [5]. Note that Ins(1,3,4)P3 and Ins(1,3,4,6)P4 are shown twice in the figure since they may originate solely from glucose or from a mixed pool, depending on the cell type and conditions.

Importantly, the deletion of either IPMK or ITPK1 greatly decreases both InsP5 and InsP6 in multiple cell lines, indicating that these enzymes cannot compensate for one another in cells, despite their multikinase activities [5,24,25]. While ITPK1 is capable of synthesizing most of the InsP repertoire and can make some unidentified version of InsP5 (not Ins(1,3,4,5,6)P5) in vitro, in mammals, IPMK activity appears to be necessary for the production of Ins(1,3,4,5,6)P5, the single IPPK substrate that is converted to InsP6 [5,26]. The apparent absolute requirement for IPMK is curious given that conditional knockout mouse models of IPMK do not have the same severity of phenotypes as downstream kinases such as IP6K1, suggesting that either an additional enzyme(s) can produce Ins(1,3,4,5,6)P5 or that InsP6 can be transported between cells, even though it is undetectable in plasma [14,27].

IPMK and InsP3K both produce Ins(1,3,4,5)P4 from Ins(1,4,5)P3, and IPMK appears to act as a redundant enzyme in this reaction since the loss of all InsP3K isoforms is dispensable for InsP4 production, at least in mouse embryonic fibroblast (MEF) cells [28]. Overall, IPMK appears to be strongly linked with the lipid-dependent branch (such as the yeast homolog Ipk2), while ITPK1 is the only enzyme known to produce InsP4 in the glucose-dependent branch. The relative proportions of the four InsP4 isomers shown in Figure 2 vary based on cell type and condition and turn over rapidly, with a “typical” steady state total cellular concentration of 3–4 µM [29]. We marked the ITPK1 product Ins(3,4,5,6)P4 in orange to highlight its unique status as an inhibitor of Ca^2+^-activated chloride channels. It is most abundant (1–10 µM) in specialized cells, including epithelial cells and neurons, and can be produced by ITPK1 through a 1-phosphatase activity or a phosphotransferase reaction (indicated by magenta arrows) [30].

InsP5 is converted to InsP6 by a single inositol-pentakisphosphate 2-kinase (IPPK) isoform in mammalian cells. InsP5 and InsP6 are the most abundant InsPs (15–50 µM) [29] but are very slowly labeled using 3H-inositol (3–5 days) [22], suggesting that only a fraction of Ins(1,4,5)P3 is directed into these pools (the contribution from the glucose-dependent branch is currently unknown). Although signaling roles have been identified for both InsP5 and InsP6, their primary role in nutrient metabolism may be to serve as stable precursor pools for the critically important PP-InsPs. In Figure 2 the PP-InsPs are indicated by the presence of a second phosphate, represented by a yellow circle, attached to the existing phosphate in the 1, 5, or 4 position. InsP6 kinases convert InsP6 to PP-InsPs such as 5PP-InsP5 (aka 5-InsP7), a crucial metabolic signal. 5-InsP7, more than the other InsP/PP-InsPs, interacts in vivo with pleckstrin homology (PH) domains—protein modules that bind to various PtdInsPs such as PtdIns(4,5)P2 and PtdIns(3,4,5)P3 and anchor proteins at subcellular membranes [31,32,33]. This competitive interaction has been proposed to increase the specificity of PtdInsP-induced signaling responses by preventing PH domain-membrane association until a threshold level of PtdInsP is reached, essentially increasing the signal-to-noise ratio by reducing background activity levels [34]. This cellular “static” may be important for fine-tuning signaling cascades in the growth factor-dependent metabolic and nutrient-sensing pathways by connecting them to energy stores. Under this paradigm, energy-replete cells (high in PH-domain interfering 5-InsP7) would require a stronger stimulus to activate, for example, the insulin signaling pathway than energy-deficient cells. 5-InsP7 can be further phosphorylated by diphosphoinositol pentakisphosphate kinase (PPIP5K) to create 1,5PP-InsP4 (aka InsP8). The level of 1,5PP-InsP4 is approximately 10-fold lower than 5-InsP7 in cells, but the impact on cell metabolism is disproportionately large. This is due in part to the ability of 1,5PP-InsP4 (and InsP7) to regulate proteins through pyrophosphorylation [35], but the main contribution may be in the control of cellular phosphate homeostasis, which is discussed in the final section in some detail.

Overall, the concept of two converging branches of InsP/PP-InsP synthesis underscores the flexible and nutrient-responsive nature of the inositol phosphate system, making it particularly suited to metabolic sensing. What remains to be determined is the extent to which the production of higher inositol phosphates (InsP5–InsP8) are dependent on each branch and how much this varies with cell type, task, and metabolic state. One roadblock to reading and understanding the cellular InsP code has been the specialized skills and equipment required to label and identify the various InsP/PP-InsPs. Fortunately, advances in technology are making the identification of stereoisomers more effective [36], and the introduction of a gel-based detection method has provided the ability to simply and routinely monitor total mass changes of the critical PP-InsPs (InsP7 and InsP8), albeit without informing on their parental sources (inositol or glucose) [37]. Overall, routine analyses of the PP-InsPs responses to metabolic challenges will vastly improve our understanding of the interplay between the inositol phosphate system and energy homeostasis, providing important insights into metabolic reprogramming.

### 2.2. The Inositol Phosphate System Coordinates Metabolic Sensing

Inositol phosphate signaling is an inherent part of cellular signaling and is instrumental in metabolic processes. As an essential nutrient, the effects of this simple polyol have been studied for many years, and a recent meta-analysis of myo-inositol supplementation trials found that inositol measurably improves the glycemic parameters of people at risk for type II diabetes mellitus [38]. While studies on the beneficial effects of inositols are often focused on endocrine effects, such as the insulin-sensitizing functions of inositol polyglycans (IPGs) [39], inositol is also a fundamental part of the cellular machinery in the form of inositol lipids and polyphosphates that participate in the cellular and systemic responses to changes in nutrient availability. These include the regulation of insulin signaling, the regulation and coordination of the AMPK and mTOR nutrient sensors, and the coordination of adaptive responses to cellular energy demands.

#### 2.2.1. Regulation of the Insulin/PI3K/AKT Axis by PtdInsPs, IPMK, and 5-InsP7

Virtually all cells have insulin receptors that transmit signals to the cell via the PI3K/AKT axis, which is the heart of the insulin signaling pathway. The key upstream event is the activation of class I phosphatidylinositol 3-kinase (PI3K) by insulin receptor substrate 1 (IRS1) for the production of PtdIns(3,4,5)P3 by PI3K and PtdIns(3,4)P2 by SHIP2, a PtdIns(3,4,5)P3 5-phosphatase [40]. These PtdInsP signals are indispensable for the activation of AKT serine/threonine kinase 1 (AKT) by two protein kinases—protein kinase D (PDK1) and mammalian Target of Rapamycin Complex 2 (mTORC2) [41]. AKT is a critical effector of the insulin signaling response, including membrane translocation of the glucose transporter GLUT4 to increase glucose uptake [42], activation of glycogen synthesis, inhibition of gluconeogenesis and fatty acid oxidation in the liver and of lipolysis in adipocytes, as well as termination of insulin release from beta cells [43].

The contribution of PI3K to insulin response is indisputable. However, a decade ago, IPMK was shown to possess PI3K activity, which was necessary for growth factor-dependent AKT activation in both mouse and human cultured cell lines [44]. Since then, the structure of human IPMK (hIPMK) has been determined, and the features that endow hIPMK with PI3K activity have been identified [45]. However, it is only recently that IPMK has been shown to regulate insulin-dependent AKT activity in vivo. Jung et al. deleted IPMK from the liver of mice and found a 50% decrease in insulin-induced AKT phosphorylation at both the PDK1 site (T308) and the mTORC2 site (S473). This defect could be rescued by the ectopic expression of IPMK or by the pharmacological activation of AKT with the agonist SC79 in hepatocyte primary cell culture. Consequently, mice lacking liver IPMK showed symptoms of hepatic insulin resistance, including reduced glycogen storage and increased gluconeogenesis in the presence of insulin, and the tendency to gain more weight than their wildtype litter mates on a high fat diet [46]. While this study shows that IPMK contributes to AKT activation in vivo, the InsP6 kinases have been found to attenuate insulin signaling in two distinct ways: by promoting insulin release and by attenuating AKT activity. The effect on insulin release was determined through an overexpression study of InsP6 kinases in murine pancreatic beta cells, which found that InsP7 was able to stimulate exocytosis of the readily-releasable pool of insulin-containing vesicles [47]. In this study, treatments that increased InsP7, including the overexpression of each of the three human isoforms of IP6K (IP6K1, IP6K2, and IP6K3), had similar stimulatory effects on exocytosis. However, only the knock down of IP6K1 reduced exocytosis, indicating that IP6K1 was the biologically-relevant isoform in mice. 5-InsP7 and InsP6 were subsequently shown to bind to the calcium sensor synaptotagmin 7 (SYT7), preventing SYT7 from binding PtdIns(4,5)P2 in unstimulated cells [48]. However, muscarinic-acetylcholine receptor-stimulated calcium release sequestered 5-InsP7, freeing SYT7 for the PtdIns(4,5)P2-dependent membrane binding to facilitate insulin vesicle release.

The effect of the whole-body gene deletion of IP6K1 in mice was subsequently found to reduce circulating plasma insulin, as predicted by the study in beta cells, yet these mice maintained normal glucose levels and glucose tolerance, characteristic of insulin hypersensitivity [49]. In addition, IP6K1^−/−^ mice fed a high fat diet were protected from many aspects of metabolic disruption typically observed in their wild type counterparts, including obesity and hyperglycemia, despite the decreased plasma insulin concentration. The insulin hypersensitivity was unexpectedly determined to be the result of increased AKT activation, suggesting the loss of a negative regulator. Attenuation of AKT activity was rescued by active IP6K1 only, and both in vitro and in vivo work have confirmed that 5-InsP7 is a competitive inhibitor of the AKT PH domain (IC50 = 1 µM), interfering with AKT membrane association [31]. It is worthwhile to note that IPMK loss also causes the loss of PP-InsPs, and it is unclear to what extent the residual AKT activity in the IPMK liver knock out model is due to class I PI3K activity or to increased AKT membrane association that follows the loss of 5-InsP7.

A conditional knock-out study that targeted the deletion of IP6K1 from adipose tissue found this was sufficient to improve metabolic parameters when the mice were subjected to minor cold stress (23C) due to enhanced thermogenesis and adipocyte browning [50]. This study identified increased LKB1-dependent AMPK activation in the knock-out animals, which was proposed to be the consequence of increased levels of (or access to) InsP6 in the IP6K1^−/−^ adipocyte knock out animals. InsP6 was found to support the LKB1 activation of AMPK in vitro in the absence of the LKB1 cofactors STRADα/MO25, although the physiologic relevance of this is unclear [50]. In contrast, the deletion of IPMK from mouse adipocytes had no effect on thermogenesis [51]. This supports the supposition that InsP6 promotes AMPK-mediated thermogenesis since the loss of IPMK decreases InsP5 (and therefore InsP6) in cultured cells [24,44]. In contrast, the knock out models of the additional IP6K isoforms have shown little overlap in the metabolic phenotype of IP6K1. For example, the whole-body loss of IP6K3, which is highly expressed in muscle but not in the pancreatic beta-cell or in the adipocytes of adult mice, produces a mild protection against age-induced weight gain that is only evident in aged mice (1–1.5 years) [51], while the whole body knock out of IP6K2 does not result in metabolic changes [52]. This is consistent with the variations in gene expression of these isoforms across tissue and cell types, with *IP6K1* as the dominant isoform in most adult metabolic tissues [53].

For obvious reasons, interest in IP6K1 is growing as a therapeutic target. A small-scale study on individuals with insulin resistance found that IP6K1 levels (protein and mRNA) deceased under conditions of high intensity exercise that correlated with improved insulin signaling, supporting an antagonistic role for insulin signaling by IP6K1 in humans [54]. While work is ongoing to create a specific IP6K1 inhibitor for the treatment of insulin resistance this may prove difficult, as IP6K1 is also important in the regulation of phosphate homeostasis [55,56], which is discussed in more detail in a later section.

#### 2.2.2. The Inositol Phosphate System Coordinates AMPK/mTOR Switching

AMPK and mTOR are two metabolic signaling hubs that exist in almost all eukaryotic organisms and are essential for the balance between energy production and utilization [57]. AMPK is the key regulator of metabolism during times of low cellular energy, and this is a direct consequence of its allosteric activation by AMP with few exceptions [57,58]. The primary role of AMPK is to promote energy production, which it does through the activation of catabolic pathways such as fatty acid oxidation, autophagy, and mitogenesis, and to inactivate energy-consuming processes such as protein synthesis and channel activities [59]. mTOR, the metabolic counterpart to AMPK, is active during times of nutrient sufficiency and stimulates processes that promote cell growth, including protein synthesis, lipogenesis, cell growth, and cell proliferation [60]. The regulation of these enzymes and their complexes have been thoroughly studied, and although there is a reliable understanding of their major regulatory interactions, refinement of these models is continually ongoing as researchers find additional connections. Over the last few decades, it has become increasingly apparent that the inositol phosphate system is a major contributor to metabolic signaling through the coordination of these regulatory nodes [19,34,61,62].

AMPK is activated in response to a variety of cellular stresses that lead to energy depletion, most specifically to a change in the ATP/AMP ratio. Each heterotrimeric AMPK complex is composed of three subunits: the catalytically active alpha subunit, and two regulatory subunits, termed beta and gamma [63]. AMPK is activated by the phosphorylation of Thr172 in the activation loop of the alpha subunit by an upstream kinase (most often LKB1), which is facilitated by the binding of AMP to the gamma regulatory subunit [64]. This allosteric regulation by AMP marks AMPK as a cellular energy sensor by promoting the activation of the catalytic alpha subunit by upstream kinases and blocking inactivation by phosphatases [59]. mTOR (mechanistic target of rapamycin) is the product of a single gene in mammals that serves as the active component of two separate protein complexes known as mTORC1 and mTORC2. These complexes are regulators of numerous processes in mammalian cells and have been reviewed in detail [64,65]. mTORC1, the complex primarily responsible for the anabolic roles of this protein, promotes protein synthesis via the activation of S6K1, lipid synthesis via sterol regulatory element binding protein (SREBP), and inhibits autophagy while AMPK protects cellular energy levels by stimulating glucose uptake, inhibiting lipid synthesis by inactivating SREBP, and priming the cell for the initiation of autophagy [57]. The subcellular localization of AMPK and mTORC1 at the lysosome is key for many of their metabolic functions, and the coordination of their antagonistic activities occurs in large part at this organelle [66]. As we outline in this section, inositol signaling plays important roles in the regulation of these enzymes.

mTORC1 and AMPK are reciprocally regulated at the lysosome through changes in the composition of a super-complex that originates at the vacuolar ATPase (V-ATPase) and is dependent on the intracellular glucose level (9 mM in fed and about 3 mM in fasted mouse liver) [67,68]. The scaffolding protein AXIN1/2 and the glycolytic sensor aldolase are key components of this system, and this finding enables the re-interpretation and consolidation of many seemingly disparate studies. In the presence of glucose, aldolase that is associated with the V-ATPase super-complex binds and retains fructose 1,6-bisphosphate (F1,6BP), the product of the aldolase reaction, which in this instance functions as an allosteric factor [69,70]. This transmits a conformational change through aldolase to the Ragulator complex that facilitates RAGG-protein activation [71]. The activated RAG proteins interact with the RAPTOR subunit of the mTORC1 complex to transiently recruit mTOR to the lysosome, where it is activated by RHEB [72]. As glucose levels decline, the glycolytic metabolite F(1,6)BP decreases, ultimately detaching from aldolase through a mass-action effect and producing a conformational change in the signaling complex that favors the association of AXIN:LKB1 with the Ragulator component LAMTOR1. This promotes the formation of the lysosomal AMPK activation complex, bridging LKB1 with lysosomal AMPK for activation [70,73]. Inositol phosphate signaling has been observed to participate in AMPK and mTOR regulation, not only by providing signaling molecules, but also through scaffolding functions of inositol phosphate multikinase (IPMK).

##### Regulation of AMPK by IPMK, 5-InsP7, and Inositol

**Figure 3:**
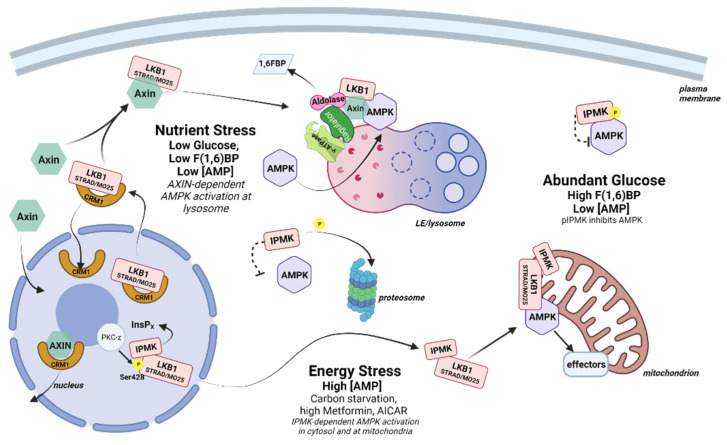
AMPK regulation by IPMK. In cells with abundant glucose, IPMK is phosphorylated and binds to AMPK, blocking activation by LKB1. When cells experience low glucose levels, IPMK is dephosphorylated, releases AMPK, and is subsequently degraded, leaving AMPK free to translocate to the late endosome/lysosome. AXIN, a nuclear/cytoplasmic shuttling protein, binds to the LKB1 complex and transports it to the lysosome, where AXIN interacts with aldolase and bridges LKB1 with lysosomal AMPK for activation. Under conditions of severe energy stress, IPMK activity is necessary to enable the LKB1 complex to exit the nucleus, possibly by facilitating the phosphorylation of LKB1 by PKCζ. The LKB1 complex is free to associate with and activate cytosolic and mitochondrially localized AMPK. Created with BioRender.com, accessed on 27 May 2022.

AMPK has been described as existing in three spatially distinct pools that are hierarchically activated based on the degree of cellular and/or energy stress—lysosomal, cytosolic, and mitochondrial (Figure 3). The lysosomal pool of AMPK responds to low glucose, while the cytosolic and the mitochondrial pools are activated by moderate (<60 µM) and high (>60 µM) concentrations of AMP [74]. Based on this model, under low glucose conditions, lysosome-associated aldolase releases bound fructose(1,6)bisphosphate (F(1,6)BP—a glycolytic metabolite), resulting in a conformational change that promotes the docking of LKB1 and the activation of AMPK in an AXIN-dependent, AMP-independent manner. During moderately severe or prolonged nutrient stress (e.g., >16 h of fasting in mice) a cytosolic pool of AMPK is activated in an AXIN-dependent, aldolase-independent manner. Finally, under extreme stress, an additional pool of AMPK associated with mitochondria becomes activated in an AXIN-independent manner.

Lysosomal AMPK complex formation is dependent on the glucose sensing function of aldolase and appears to occur before measurable changes in the intracellular AMP/ATP ratio (at least with commonly used methods) [74]. This is consistent with a fine-tuning function for AMPK in energy preservation that varies with tissue and cell type. In the hypothalamus, AMPK is extremely responsive to glucose variations, which is important for the hypothalamic regulation of whole-body energy balance, including food intake, thermogenesis and glucose homeostasis [75]. This sensitivity has been shown to be coordinated in part through an activity-independent interaction with IPMK. Bang and coworkers observed that *IPMK* gene expression and protein levels were low in the hypothalamus of fasted mice (24 h) and increased substantially within 30 min of refeeding, inversely correlating with AMPK activation. MEF cells that had been carbon-starved in Krebs buffer for 2 h also exhibited high pAMPK levels, and these rapidly decreased once glucose was restored. In contrast, MEF cells depleted of IPMK (IPMK^−/−^) maintained high pAMPK levels despite the restoration of glucose, suggesting the loss of a negative feedback signal. This negative feedback was determined to be through the association of AMPK with the IPMK protein under high glucose conditions. IPMK pull-down experiments identified a complex containing IPMK, LKB1, and AMPK in the presence of glucose that was disrupted following glucose withdrawal in both MEF cells and hypothalamus-derived GT1-7 cells. The association of IPMK with AMPK was dependent on the enhanced tyrosine phosphorylation of IPMK at Tyr174 by an unidentified kinase. In addition, both IPMK mRNA and protein are depleted during glucose starvation in MEFs and during fasting in the hypothalamus and arcuate nucleus of conditional knock out mice, reinforcing the connection to metabolic function [26].

In a second study by this group, the interaction between IPMK and AMPK was investigated in cells that were first cultured under low glucose conditions, then subsequently treated with high concentrations of the AMPK activator AICAR (2 mM) or metformin (4 mM)—treatments that have now been shown to simulate extreme stress conditions in MEF cells [74]. Contrary to the previous findings, under these more stressful conditions, loss of IPMK inhibited rather than activated AMPK. Loss of IPMK was correlated with the decreased phosphorylation of LKB1 on S428, which, along with pS307, has been shown to be necessary for LKB1 to exit the nucleus [76,77], suggesting that IPMK is necessary for LKB1 nuclear export. This phenotype was rescued both by the expression of catalytically active IPMK and by the substitution of atIPK2b, an IPMK homolog from *Arabidopsis thaliana* that lacks the PI3K activity observed in mammalian IPMK. Further, the expression of a catalytically inactive form of IPMK did not rescue AMPK activation, confirming the need for a soluble InsP/PP-InsP. Subsequent deletion mapping and peptide competition experiments showed that IPMK also served a bridging function to promote AMPK activation by LKB1 [24].

Together, these data indicate that IPMK can serve in both inhibitory and stimulatory capacities in AMPK signaling dependent on the severity of cellular stress (Figure 3). During times of high energy (low AMP), pIPMK(Y174) binds to AMPK, perhaps preventing AMPK association with the lysosome or with activating kinases. When glucose levels fall, IPMK is dephosphorylated and degraded, releasing AMPK for activation at the lysosome by LKB1 in an AXIN/aldolase-dependent manner. Once the glucose level is restored, the expression of the IPMK protein promotes AMPK inactivation by competing with LKB1 for AMPK binding. In addition, glucose metabolism restores energy levels, leading to an increase in InsP7 (see Section 2.3 below) that promotes LKB1 retention in the nucleus, preventing further activation of AMPK by LKB1 [78]. Under conditions of extreme stress, the production of an unidentified InsP by nuclear IPMK results in (PKC-ζ mediated) LKB1 release from the nucleus, with IPMK serving as an LKB1 chaperone to facilitate the AXIN-independent activation of AMPK in the cytosol and/or at the mitochondria. In terms of AMP levels, when the level of AMP is low (for example, in the “fed” state), IPMK acts as a brake on AMPK signaling that is released as AMP levels increase (for example, in the “fasted” state). However, under conditions of high AMP associated with extreme cellular stress this role is reversed, and nuclear IPMK promotes AMPK activation by facilitating the interaction of LKB1 and AMPK.

Recently, myo-inositol itself was shown to compete with AMP for binding to the gamma subunit (the AMP sensor) of AMPK, acting as an endogenous AMPK suppressor [79]. When the cellular AMP concentration is low, the AMP/inositol ratio facilitates binding of inositol to AMPKγ. However, when cells are deprived of nutrients and AMP levels begin to rise, the increase in the AMP/inositol ratio causes the displacement of inositol by AMP, leading to the activation of AMPK. While tissues within an organism are rarely exposed to glucose starvation, intracellular myo-inositol levels are sensitive to regulation at multiple points, contributing to the activation of AMPK in unexpected ways. Just as AMPK is influenced by inositol signaling, inositol signaling is influenced by AMPK. For example, AMPK phosphorylation of FYVE domain-containing phosphatidylinositol 3-phosphate 5-kinase (PIKFYVE) stimulates the conversion of PtdIns3P to PtdIns(3,5)P2 on endosomes to promote GLUT4 membrane translocation and glucose uptake in skeletal muscle glucose uptake [80]. It is worth noting that AMPK phosphorylates the same residue on PIKFYVE that is targeted by AKT following insulin stimulation, showing a rare convergence of AMPK and AKT signaling [81].

Overall, AMPK undergoes multiple negative checks from the inositol phosphate system, underscoring the dominance of inositol signaling in growth factor pathways. A clearer understanding of the sub-pools of AMPK and their responses to different stress signals will improve our understanding of crosstalk between inositol phosphates and AMPK signaling.

##### Regulation of mTORC1 and mTORC2 by PtdInsPs and IPMK

Inositol signaling intersects with mTORC1 and mTORC2 at multiple points and in multiple subcellular locations. Both complexes are recognized nutrient sensors: mTORC2 is tied to the redox levels in the cell through reversible acetylation that modulates its activity [82], and mTORC1 is sensitive to the convergence of growth factors and amino acid levels. Inositol phosphate signaling controls both negative and positive aspects of mTORC1 signaling. RHEB GTPase, the primary activator of mTOR kinase, allosterically activates mTORC1 on the lysosome when RHEB is in the GTP-bound state. RHEB activation is regulated by the tuberous sclerosis protein complex (TSC), a component of the larger TSC2 complex. The intrinsic GTPase activity of RHEB is low, and the GAP activity of TSC is critical to maintain RHEB in the GDP-bound form to prevent the activation of mTORC1. RHEB is a prenylated protein that is constitutively associated with endomembranes, including the lysosome, while TSC2 retention on the lysosome is maintained through interactions with RHEB-GDP and lysosomal PtdIns(3,4)P2 [83,84,85]. Although TSC is crucial for the inhibition of mTOR activity, the association between TSC and RHEB must be released before mTOR can be activated, and this is dependent on the growth factor-induced activation of AKT. Phosphorylation of TSC2 by AKT initiates the re-localization of the TSC complex from the lysosome to unidentified subcellular puncta in a process that requires the production of PtdIns(3)P and PtdIns(3,4)P2 at the lysosome for AKT recruitment [86,87]. Note that PtdIns(3,5)P2 has also been proposed to act as a recruitment factor for mTORC1 substrates in yeast, including ribosomal protein S6 kinase (S6K, yeast Sch9), the mTORC1 effector that increases protein translation [88]. This is an appealing model as it reconciles the apparent need for TORC1 to translocate to lysosomes to be activated, when many of its effectors are cytosolic proteins. Interestingly, both the yeast and mammalian forms of the RAPTOR scaffolding protein (Kog1 in yeast) have been discovered to bind PtdIns(3,5)P2 in vitro, suggesting that RAPTOR may also respond to PtdIns(3,5)P2 to guide mTORC1 membrane association [88].

**Figure 4:**
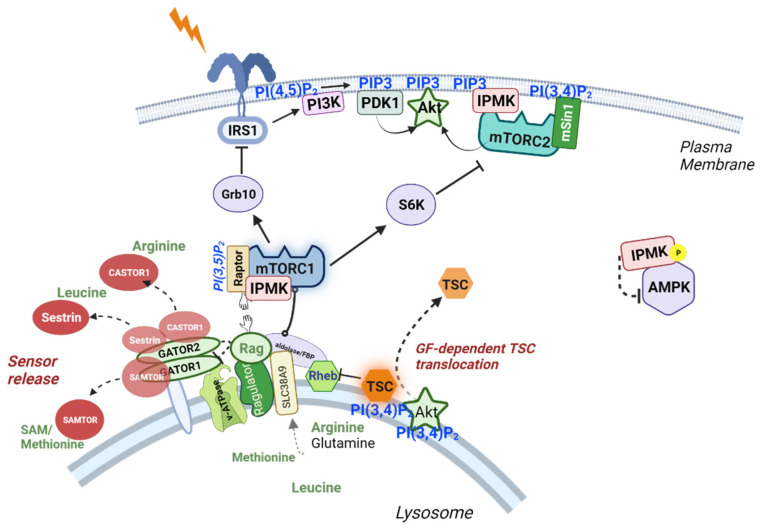
Regulation of mTORC1 and mTORC2 by IPMK and PtdInsPs. A representation of the inositol phosphate components that interact with mTORC1 at the lysosome, including PI(3,4)P2 recruitment of Akt to inactive TSC and PI(3,5)P2 interaction with RAPTOR for membrane association, and IPMK interaction to stabilize mTORC1 association with the RAG GTPase proteins at the Ragulator complex. Binding of arginine and leucine to the amino acid sensors CASTOR1 and Sestrin cause them to release from the lysosomal surface, providing room for mTORC1 to dock with RAG proteins for activation by RHEB. Separately, mTORC2 interacting with IPMK associates with PI(3,4)P2 through the mSin1 PH domain, while IPMK produces PI(3,4,5)P3 for the activation of Akt and PDK1. Activation of the insulin receptor (represented by a lightning bolt) also stimulates PI3K to produce PIP3 for PDK1 and Akt activation. Under these nutrient-rich conditions, a pool of cytosolic IPMK binds and prevents the activation of AMPK. Created with BioRender.com, accessed on 27 May 2022.

In order to be activated, mTORC1 must translocate from the cytosol to the lysosome, where RHEB, its activator, is located. The translocation of mTORC1 to the lysosome has long been known to depend on amino acid levels [89], and this function was shown to be facilitated by IPMK [90]. This translocation depends on the successful detection of branched chain amino acids, particularly arginine and leucine, by sensing proteins. In the absence of amino acids, the Sestrins (which bind leucine) and CASTOR1/2 (which bind arginine) associate with the lysosome and prevent mTORC1 from docking with the lysosomal Ragulator complex [90]. Upon binding amino acids, Sestrin and CASTOR dissociate from the lysosome, freeing the Ragulator for the RAPTOR-mediated docking with mTORC1 (Figure 4. In MEF cells, a 10-min pulse of leucine or arginine produced robust mTOR:RAPTOR association and downstream S6 kinase activity, which decreased by approximately 60% in IPMK^−/−^ cells [90].

This effect was also confirmed in HEK293T cells, where it was determined that the loss of the IPMK protein (but not catalytic activity) reduced the association between mTOR and RAPTOR in the presence of leucine or arginine, while the loss of IPMK had no effect on the mTOR:RAPTOR interaction in cells deprived of amino acids (and therefore unable to associate with Ragulator); together, these data suggest that IPMK supports the cohesion of the core mTORC1 complex during docking at the lysosome [89]. In the same study, the N-terminus of IPMK was then shown to directly interact with mTOR kinase, suggesting the possible association of IPMK with both the mTORC1 and mTORC2 complexes, and the C-terminal portion of IPMK containing the catalytic domain was shown to bind to RAPTOR, suggesting that IPMK may act as a bridging protein to stabilize the mTOR:RAPTOR association. Consistent with this conclusion, overexpression of a GFP-tagged N-terminal IPMK peptide disrupted the association between mTOR and RAPTOR in WT cells, while the expression of WT IPMK, but not IPMK lacking the N-terminal 29 amino acids, rescued the compromised mTOR:RAPTOR interaction in IPMK^-/-^ MEF cells. The connection between IPMK and mTOR determined in these studies has since been observed in unrelated work on endocannabinoid receptor signaling, which found that IPMK was required for endocannabinoid receptor-induced mTORC1 activity in MEF cells, in the fungi *Dictyostelium discoideum* [91], as well as in primary lymphocytes from multiple sclerosis patients [92], thereby supporting a role for IPMK in mTORC1 signaling.

**Figure 5:**
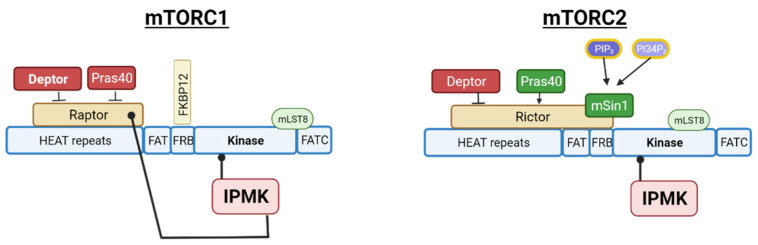
Diagram of mTORC1 and mTORC2 interactions with IPMK. Schematic diagrams of the mTOR kinase regions with key components of the mTORC1 and mTORC2 complexes. Physical associations of IPMK N-terminus with the mTOR kinase domain and the C-terminus of IPMK with Raptor are indicated by black lines. Created with BioRender.com, accessed on 27 May 2022.

As previously mentioned, the observed interaction between IPMK and mTOR kinase indicated a likely interaction of IPMK with the mTORC2 complex. mTORC2 is a multi-subunit protein complex that contains three proteins unique to mTORC2—RICTOR, mSIN1 and PROTOR1/2 [93,94,95]. In the same study on IPMK:mTORC1 associations, IPMK was confirmed to associate with mTORC2 by the IPMK pull-down of the RICTOR subunit, and these associations are shown diagrammatically in Figure 5.

While the association of IPMK with mTOR and RICTOR was not dependent on IPMK activity, the requirement for functional IPMK on mTORC2 activity has not been investigated. This is worth investigating since it may provide insights into the dependence of AKT activation on IPMK versus class I PI3Ks. One would imagine that it should be simple to address this question using PI3K inhibitors. However, while immunoprecipitated IPMK is impervious to the common PI3K inhibitors wortmannin and Ly294002 in an in vitro assay, the pretreatment of cells with these inhibitors prior to the immunoprecipitation of IPMK results in the significant inhibition of its PI3K activity, indicating some dependence on class IPI3K for its activation [44]. This makes it technically difficult to determine whether AKT activation is dependent on IPMK-produced PInsP3 as well as PI3K-generated PInsP3. Indeed, it is possible that PI3K and IPMK cooperate to fully activate AKT by individually regulating PKD (a PI3K target) and mTORC2 (a possible IPMK target), and the association of IPMK with the mTORC2 complex provides a potential mechanism for the co-localization of these enzymes. Alternatively, IPMK-generated PtdIns(3,4,5)P3 may be necessary to relieve the Sin1-mediated auto-inhibition of mTORC2 kinase activity, although it is probably not necessary for mTORC2 membrane localization, based on compelling evidence for the constitutive localization of mTORC2 at the plasma membrane in cultured cell models [96,97]. Future studies examining the requirement for IPMK activity on other mTORC2 substrates besides AKT will be helpful in addressing this question, as would any apparent dependence on IPMK activity for mTORC2 localization. Overall, multiple layers of PtdInsP, InsP, and PP-InsP signaling are necessary for the regulation of AMPK and mTOR complexes, presenting a clear illustration of metabolic coordination by inositol phosphate signaling.

### 2.3. The Inositol Phosphate System Regulates Energy Balance

Cellular energy is the single most important requirement for cell survival, and cells have evolved a robust network of signals and sensors to report on energy levels. Observed changes in glycolysis, mitochondrial morphology, and/or respiratory activity following the loss of IPMK, IP6K, and PPIP5K in yeast and mammalian systems underscore the importance of inositol phosphate signaling in mitochondrial function. Cells maintain a balance between glycolysis and respiration (oxidative phosphorylation—OXPHOS) to meet both carbon and energy requirements that fluctuate with nutrient availability, developmental state, and the overall microenvironment [98]. In mammals, most cells are in a quiescent (non-proliferative) state with low biosynthetic demands, producing the majority of cellular ATP through OXPHOS. Growth factor-stimulated increases in glucose uptake result in matching increases in OXPHOS as the product of glycolysis (pyruvate) is transported into mitochondria for additional oxidation through the Tricarboxylic Acid (TCA) cycle and respiratory chain (RC), producing ATP and consuming ADP, Pi, and oxygen. Cells with limited oxygen availability (such as tumor cells) increase glycolytic flux to satisfy energy needs, while cells that are proliferating or engaged in processes that require a net synthesis of proteins, lipids, or DNA increase glycolytic flux to provide the necessary building blocks for biosynthesis. The machinery for switching between glycolysis and OXPHOS is inherent in almost all cells (red blood cells are a notable exception) and is an essential part of cellular reprogramming that can be triggered in response to internal and external cues. Processes such as stem cell differentiation, epithelial to mesenchymal transition, and T-cell activation all require metabolic reprogramming as part of their cellular program.

Inositol phosphate signaling is an integral part of energy homeostasis and contributes to cellular energy not only by regulating growth factor signaling, nutrient uptake, and metabolic signaling as previously discussed, but through metabolic programming in unexpected ways. Numerous reports indicate that disrupting inositol phosphate synthesis impacts metabolic functions in unicellular and multicellular organisms, with effects ranging from enhanced glycolysis and gluconeogenesis to lipid synthesis, to changes in mitochondrial function, with severity dependent on cell type and changes in the InsP code [46,99,100,101,102]. For example, the genetic deletion of Kcs1 (IP6K1) in *S. cerevisiae* increased cellular ATP 3-fold and resulted in a highly glycolytic Warburg phenotype. The same result was found following the knockdown of IP6K1 in MEF cells, suggesting a conserved role for IP6K1 in regulating the metabolic program [99]. Both yeast and human cellular systems displayed reduced mitochondrial function, a decrease in mitochondrial mass, as examined by MitoTracker Green dye staining, and reduced mitochondrial membrane potential (ΔΨM), consistent with decreased respiratory activity, and Kcs1^−/−^ cells were unable to grow on substrates that required cellular respiration (e.g., ethanol, glycerol, and galactose). Finally, while cellular ATP levels were high, both cell types showed reduced proliferation rates. In the case of Kcs1 knock out cells, the increase in glycolysis was traced to the upregulation of glycolytic gene expression resulting from decreased pyrophosphorylation of the master glycolytic transcription factor GCR1, revealing feedback regulation by InsP7 attenuates glycolysis in *S. cerevisiae*. While overexpression of a dominant negative GCR1 reduced the glycolytic phenotype, the correction of the respiratory defect was not reported, so it is unclear whether the two phenomena are related [99]. The cause of the Warburg effect in IP6K1 MEF cells was not pursued and remains undetermined. However, a separate study examining the effect of IPMK on angiogenesis reported an increased expression of hypoxia inducible factor 1a (HIF1A) following IPMK deletion [103]. HIF1A is a constitutively expressed protein, but normally only accumulates under hypoxic conditions due to its rapid ubiquitinoylation and degradation. It is a well-known activator of glycolysis that is necessary to shift cells away from mitochondrial respiration during oxygen depletion. IPMK^−/−^ MEF cells showed a de-repression of multiple glycolytic genes (including phosphoglycerate kinase 1 (PGK1), pyruvate dehydrogenase kinase 1 (PDK), Solute carrier family 2, facilitated glucose transporter member 3 (SLC2A3), and L-lactate dehydrogenase A chain (LDHA)), suggesting that, such as IP6K1, IPMK also contributes to the suppression of glycolysis [103]. In this case, the mechanism involved InsP5, which was necessary to promote binding between the E3-ubiquitin ligase von Hippel Lindau factor (pVHL) and HIF1A to target HIF1A for degradation. Since IP6K1 has not been reported to inhibit IPMK activity or InsP5 production, these studies indicate that multiple components of the inositol phosphate system are involved in the regulation of energy production.

Surprisingly, a double knock-out of IP6K1/2 in the human cell line HCT116 revealed no change in mitochondrial morphology, respiration, or membrane potential, in contrast to the IP6K1 loss in MEF cells [104]. These cells did display an increase in glycolysis and ATP level and both InsP7 and InsP8 were essentially eliminated (as detected by 3H-inositol labeling), suggesting a possible negative inhibition of glycolysis by InsP7/InsP8, similar to that observed in yeast. Ablation of PPIP5K in the HCT116 cell line (which eliminates InsP8, but not InsP7) produced a new, hypermetabolic phenotype with increases in mitochondrial mass, respiration, and glycolysis. Treatment of PPIP5K^−/−^ cells with (N2-(m-trifluorobenzyl), N6-(p-nitrobenzyl)purine) (TNP), a pan-IP6K inhibitor, or a double knock out of PPIP5K and IP6K2 (which eliminates InsP7 and InsP8) did not alter the phenotype, implicating InsP8 in the metabolic reprogramming. The dependence on InsP8 for the suppression of glycolysis was repeated after depleting PPIP5K in HEK293T cells, but the increase in mitochondrial mass and respiration were not, indicating a cell-specific effect on mitochondria [105]. While the loss of InsP8 appears to reproducibly increase glycolysis, the effect on mitochondrial biogenesis (observed in the colon cancer cell line) is not reproduced in the human embryonic kidney cell line and, therefore, is likely a more indirect effect of InsP8 loss. Combined with other studies that show preferential changes in either glycolysis or respiration following loss of InsP7 and/or InsP8, it appears that these systems are dependent on pyrophosphates to different degrees, consistent with the cellular need for fine control of these primary energy pathways [34].

The similar effects of PPIP5K deletion on energy pathways in multiple organisms reinforces the evolutionary role of PP-InsPs in energy homeostasis. For example, Vip1 (PPIP5K) deletion in the yeast model *C. albicans* results in the same hyper-glycolytic and low respiration phenotype that was detected in the *S. cerevisiae* Ksc1^−/−^ (IP6K1) model; however, in *C. albicans*, the mitochondria retained functionality, and simply switching from glucose to galactose (a non-fermentable carbon source) resolved many of the observed phenotypes [100]. Note that *C. albicans* does not have the same glucose repression system that is present in *S. cerevisiae* [106], and the authors proposed that repression of respiration was due to an altered redox balance. The metabolic defect was ultimately traced to the loss of mitochondrial aconitase expression in the absence of Vip1 (PPIP5K), and ectopic expression of the missing enzyme produced a Warburg-reversing effect, restoring the balance between glycolysis and OXPHOS to a near wild-type level. This study expands the role of PP-InsP signals in the metabolic gene regulation and highlights their potential to regulate metabolic flux.

Metabolic reprogramming has also been observed in the parasite Trypanosome Brucei after the depletion of IPMK, implicating inositol phosphate signals in the developmental switch between the proliferating long slender form and the non-proliferating short stumpy form. In the absence of IPMK, the glycolysis-dependent long slender form initiated a switch to OXPHOS, a state typically only observed in the procyclic form that develops after transmission to the fly. This was accompanied by increased ATP levels and the activation of the state-specific transcriptional program for the short stumpy form [101], and it is one of the few systems that show inositol phosphate-dependent metabolic switching between glycolysis and OXPHOS cleanly. It also suggests that inositol phosphates may be important for the metabolic remodeling that occurs during cell differentiation and immune cell activation [107,108,109,110], as well as coordinating the on-demand energy shifts in committed and quiescent cells in cooperation with the AMPK and mTOR systems.

Although the systems examined are disparate, one common factor shown to contribute to metabolic disturbance is the loss of inositol pyrophosphates—in particular 5-InsP7 and InsP8 (PP-InsP4). One important consequence of decreased PP-InsP is a change in level of intracellular inorganic phosphate. Inorganic phosphate (Pi) is critical for ATP production as well as for the biosynthesis of cellular nucleotides, protein, and phospholipids. While sufficient Pi is a necessity, Pi overload can result in disrupted signal transduction and mitochondrial dysfunction [111,112,113,114]. Microorganisms such as yeast have a Pi regulatory network consisting of extracellular and intracellular Pi-sensing proteins that cooperate to adapt to phosphate levels, and mammals rely on the sodium-dependent phosphate transporters (members of the solute carrier family SLC34) to import Pi and on the Xenotropic and Polytropic Retrovirus Receptor 1(XPR1) protein for Pi efflux out of the cell [115]. Phosphate homeostasis is essential in all organisms, and PP-InsPs have been maintained across eukaryotes as fundamental regulatory components of these systems.

The connection between inositol pyrophosphates and phosphate sensing was first identified in yeast, with later work demonstrating that 5-InsP7 is essential for inorganic polyphosphate synthesis in *S. cerevisiae* [116,117] and for Na/Pi transport in *Trypanosoma brucei* [118], while InsP8 regulates Pi efflux in human cells [119]. The connection of PP-InsPs to phosphate homeostasis is even evident in the initial mis-identification of IP6K2, which was first cloned as a mammalian intestinal phosphate transporter (PiUS—Phosphate inorganic Uptake Stimulator) that stimulated the cellular uptake of Pi in *Xenopus* oocytes and was only later determined to be an inositol kinase [120]. Loss of InsP8 signals the cell that the intracellular phosphate level is low, inducing cellular responses that increase intracellular Pi levels [121]. While Pi availability drives ATP production in both glycolysis and OXPHOS, the impact of increased intracellular Pi on the described metabolic reprogramming is unclear. It is interesting to note that HEK293 cells depleted of mitochondrial polyP through the stable expression of polyP hydrolyzing enzyme scPPX exhibit reduced OXPHOS and elevated glycolysis, and this may be a contributing factor to metabolic reprogramming under some conditions [122].

Phosphoinositides should not be overlooked as regulators of bioenergetic switching, and the disruption of these may contribute to the observed defects in mitochondrial function seen after the depletion of InsP-kinases. For example, the PTEN-like mitochondrial PtdIns(3,4,5)P3 phosphatase (PTPMT) is associated with the inner mitochondrial membrane, where it plays a critical role in priming mitochondria for the rapid metabolic transition required to support the extreme energy demands of hematopoietic stem cell (HSC) differentiation. Loss of PTMPT appears to disrupt the switch from glycolysis to OXPHOS in murine HSCs through the accumulation of mitochondrial PtdInsPs, which interact with and increase Uncoupling Protein2 (UCP2) activity [123]. UCP2 is known to inhibit glucose-derived pyruvate oxidation in the mitochondria [124], and the resulting energy imbalance arrests HSC differentiation. In addition, PtdIns(4,5)P2 on the mitochondrial outer membrane recruits PKCα, which serves an established anti-apoptotic role at this compartment. Mitochondrial PtdIns(4,5)P2 depletion is necessary to initiate mitophagy, and loss or masking of mitochondrial PtdIns(4,5)P2 is sufficient to induce mitochondrial fragmentation and mitophagy in HepG2 and NIH 3T3 cells [87]. AKT, a protein dependent on PtdInsP lipids for activation, also has well studied impacts on mitochondrial function and metabolic programming. The AKT2 isoform rapidly translocates to mitochondria, following insulin growth factor and insulin stimulation, where it has a number of target proteins, including pyruvate dehydrogenase kinase (PDK). Activation of PDK by AKT2 reduces mitochondrial oxidation of pyruvate, suppressing respiration and supporting a switch to glycolysis in response to stress and hypoxia [125,126]. While there is no published data on the effect of InsP7/InsP8 loss on phosphoinositide synthesis, as there is with inositol starvation, loss of InsP7 may increase glucose-derived inositol synthesis by promoting the expression of MIPS. Perhaps more significantly, the loss of the PH-domain attenuating effects of InsP7 may alter a variety of enzyme activities, with far-reaching effects on the metabolic response.

## 3. Summary

Inositol signaling is involved in essentially all energy processing decisions made by cells from receptor engagement to energy production, and resource recycling and is instrumental in the control of energy homeostasis. Targeted disruption of this system can have profound effects on the metabolic activities of cells. Cells contain several energy and nutrient-sensing systems. While the most well-studied of these are the AMPK and mTOR systems, it has become increasingly evident that the inositol phosphate system plays a key role at the cellular level, through the regulation of AKT, AMPK, and mTOR signaling, energy sensing, and phosphate homeostasis, and at the systemic level through the regulation of insulin release and glucose sensing in the hypothalamus. Overall, the inositol phosphate system is part of a coordinated metabolic response that encompasses growth factor signaling, nutrient sensing, and the maintenance of cellular energy stores. The turnover of phosphoinositides not only alters the levels of PtdIns(4,5)P2 and PtdIns(3,4,5)P3, but it also impacts the relative levels of InsP/PP-InsPs. This creates a dynamic inositol phosphate code, which sets into motion a necessary and indispensable network for communicating and coordinating information about the availability of nutrients to orchestrate cellular responses that balance energy supply with demand. Due to the profound influence of the inositol phosphate system on the coordination of nutrient sensing and bioenergetic pathways, we propose that this system should be viewed as a single network with respect to nutrient metabolism and bioenergetic homeostasis.
